# Changes in Physicochemical Properties and Volatile Compounds of Roselle (*Hibiscus sabdariffa* L.) Calyx during Different Drying Methods

**DOI:** 10.3390/molecules26206260

**Published:** 2021-10-16

**Authors:** Nurul Hanisah Juhari, Helle Jakobe Martens, Mikael Agerlin Petersen

**Affiliations:** 1 Department of Food Service and Management, Faculty of Food Science and Technology, Universiti Putra Malaysia, Serdang 43400 UPM, Selangor, Malaysia; 2 Section for Forest, Nature and Biomass, Department of Geoscience and Natural Resource Management, University of Copenhagen, Rolighedsvej 23, DK-1958 Frederiksberg, Denmark; hjm@ign.ku.dk; 3 Design and Consumer Behaviour, Department of Food Science, Faculty of Science, University of Copenhagen, Rolighedsvej 26, DK-1958 Frederiksberg, Denmark; map@food.ku.dk

**Keywords:** roselle (*Hibiscus sabdariffa* L.), drying, dynamic headspace sampling, physicochemical properties, volatile compounds, microstructure

## Abstract

Fresh roselle are high in moisture and deteriorate easily, which makes drying important for extending shelf-life and increasing availability. This study investigated the influence of different drying methods (oven-drying, freeze-drying, vacuum-drying, and sun-drying) on the quality of roselle calyx expressed as physicochemical properties (moisture content, water activity, soluble solids, color), volatile compounds, and microstructure. Oven-drying and freeze-drying reduced moisture content most while vacuum-drying and sun-drying were not as efficient. All drying methods except sun-drying resulted in water activities low enough to ensure safety and quality. Vacuum-drying had no impact on color of the dry calyx and only small impact on color of water extract of calyx. Drying reduced terpenes, aldehydes, and esters but increased furans. This is expected to reduce fruity, floral, spicy, and green odors and increase caramel-like aroma. Sun-drying produced more ketones, alcohols, and esters. Scanning electron microscopy revealed that freeze-drying preserved the cell structure better, and freeze-dried samples resembled fresh samples most compared to other drying techniques. The study concludes that freeze-drying should be considered as a suitable drying method, especially with respect to preservation of structure.

## 1. Introduction

Roselle (*Hibiscus sabdariffa* L.) locally known as *asam belanda*, *asam susur* or *asam paya* in Malaysia, is a member of the Malvaceae family. It is originally native from India to Malaysia, but now it is widely distributed and cultivated in tropical and subtropical regions all around the world. Many parts of roselle including seeds, leaves, fruits, and roots are used in various foods. Among all, calyces are the most popular being used for making soft drinks, juices, teas, wines, jams, jellies, pickles, fruit leathers, yogurts, and colorants. Overseas demand for roselle products is also very encouraging [1,2]. In addition to the increasing popularity of roselle in healthcare, beverages, and cosmetics [3], roselle is a plant which can be used to improve the quality of food products. Calyces are rich in vitamin C, minerals and other antioxidants such as anthocyanin, flavonoids [4], and phenols [5], which are hypothesized to be beneficial to human health by preventing cancer and reducing chronic illnesses such as diabetes, dyslipidemias, high blood pressure, and coronary heart disease [6].

Drying is one approach that can be applied to prolong the shelf-life of fresh materials. Several commercial drying techniques are available, and every type has its own advantages and limitations and results in different nutritional and physicochemical characteristics of the final product. In addition, a good drying technique can enhance the quality of the product significantly [7]. Various drying techniques such as spray-drying [8], freeze-drying [9], solar thermodynamic drying [10], fixed bed drying [11], sun-drying, and oven-drying [12] have been applied to roselle. For dried roselle, the aroma and flavor are affected by the drying parameters and drying conditions [13]. Mostly, drying methods assure microbial stability, guarantee shelf-life of the product, and facilitate packaging and distribution [7,14,15]. Agudelo et al. [16] showed that for the manufacture of powered fruit, freeze-drying method is highly recommended. Furthermore, Martínez-Navarrete et al. [17] showed that freeze-drying is one of the methods that better preserves the bioactive compounds of the fruits, but, unfortunately, is very expensive. Therefore, studies of different drying methods are needed in order to attain the desired quality of the final product.

To our knowledge, no studies have been done to evaluate the influence of different drying methods on roselle calyx in terms of physicochemical properties, volatile compounds, and microstructure in comparison with the fresh roselle. Thus, the objective of this study was to determine the influence of oven-drying, freeze-drying, vacuum-drying, and sun-drying on the physicochemical properties, volatile compounds, and microstructure of roselle (*Hibiscus sabdariffa* L.). This study would be considered valuable when selecting appropriate drying methods for roselle with the aim of fulfilling the consumer demand for processed food products that are close to fresh samples and retain more of the original characteristics. Due to the high moisture content of fresh roselle, it is vital to produce a high-quality dried roselle calyx.

## 2. Results and Discussion

### 2.1. Physicochemical Analysis

Table 1 shows that there were significant (*p* < 0.001) differences in all physicochemical properties measured. Fresh roselle calyx contained 89.4% of moisture (water activity 1.000). Sun-drying turned out to be the least efficient drying method since sun-dried samples had the highest moisture content (15.6%, water activity 0.727). This result was due to the weather because a haze phenomenon occurred in Malaysia from September until November 2015. The entire process of sun-drying was disturbed since the haze prevented the penetration of sunlight to the samples. All oven and freeze-dried samples had moisture contents below 10% (water activities 0.395–0.482). Materials with excessive moisture content will be susceptible to bacteria and molds but since water activity was 0.605 or lower in oven and freeze-dried samples, they were considered safe for general storage [18,19].

Extracts based on freeze-dried roselle had the highest value of total soluble solids (TSS) (Table 1). A higher value of TSS may be attributed to longer drying time and possibly pectic enzyme activity [20] which may increase the amount of soluble solids. Other factors could be better structure of the dried product due to higher porosity at lower shrinkage, which enhances the grinding process and thus extraction.

Color is an important quality attribute in roselle; in fact, roselle is used as food coloring due to the high content of anthocyanin. In this study, the effects of different drying methods on the color of dried roselle calyx were measured in ground calyces and in a liquid extract (‘filtrate’). Preferred colors are those closest to the original color of fresh roselle. Color produced by ‘FRESH’ was considered the reference color both in ground and liquid form. In both ground and liquid form, the color of roselle produced by vacuum-drying was closest to the color of fresh roselle (Table 1). This is most probably related to vacuum-drying being based on the rule of producing a vacuum to reduce pressure below vapor pressure of the water and then pressure is decreased around the samples to be dried. During oven-drying and sun-drying, samples were exposed to a continuous flow of hot steam or air [21] which led to discoloration and degradation of the material. Different intensities in red color were observed between drying methods. In liquid form, ‘FRESH’ and ‘VACUUM’ produced light red-yellow color while ‘FREEZE’, ‘OVCOM’, ‘OVLAB’, and ‘SUN’ produced dark red-blue color. However, in ground form, ‘FREEZE’, ‘OVCOM’, and ‘OVLAB’ produced more lighter red-yellow than ‘FRESH’, ‘VACUUM’ and ‘SUN’ (dark red-yellow). Furthermore, in ground form, freeze-dried roselle calyx had higher redness while sun-dried roselle calyx had higher yellowness. However, in liquid form, ‘FRESH’ was highest both in redness and yellowness. Results related to color of the dried roselle are in accordance with those reported by Juhari et al. [22] and Prachayawarakorn et al. [23], who reported that the main factors affecting color change of materials during drying process were drying time, drying temperature, and loading capacity. The color of roselle that was measured in liquid form showed similar tendencies (Table 1). The processes behind the color changes can be breakdown of anthocyanins, change in anthocyanin color caused by changes in pH, Maillard reactions, and caramelization [24,25].

### 2.2. Volatile Compounds

A total of 74 volatile compounds, consisting of terpenes (18), esters (16), aldehydes (12), ketones (11), furans (6), alcohols (6), phenols (3), an acid (1), and a lactone (1) were identified in the samples (Table 2). The major volatile compounds (by peak size: methyl acetate, ethyl acetate, 2-butanone, 2-methylbutanal, 3-methylbutanal, pentanal, hexanal, heptanal, methyl hexanoate, limonene,1-pentanol, p-cymene, α-terpinolene, octanal, 2,6,6-trimethylcyclohexanone, 6-methyl-5-hepten-2-one, nonanal, isoterpinolene, furfural, benzaldehyde, linalool, phenylethanal,1-phenylethanone, α-terpineol, azulene, δ-cadinene, p-cresol, α-calacorene, and phenol) were found in all samples, but in varying levels.

**Figure 1:**
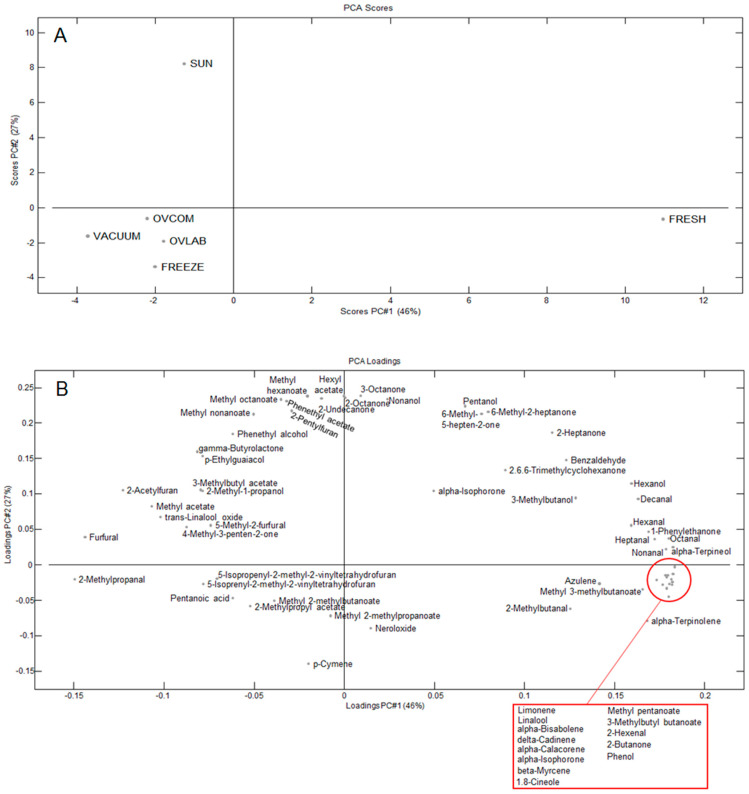
PCA (**A**) scores and (**B**) loadings plots of based on average significant (*p* < 0.05) peak areas of volatile compounds for fresh and dried roselle (*Hibiscus sabdariffa* L.) calyx samples.

A Principal Component Analysis (PCA) was carried out using the peak areas relative to dry matter content exhibiting significant (*p* < 0.05) variation (Figure 1). The first principal component (PC1) explained 46% of the variance while PC2 explained 27% of the variance. Overall, ‘FRESH’ is rather different from dried samples, regardless of the drying method, being characterized by high levels of many terpenes and some aldehydes and esters (mainly branched chain). Terpenes are typical plant volatiles. Twelve of the 18 detected terpenes were significantly higher in ‘FRESH’ and they have odor descriptors like fruity, floral, pine/woody, and spice (Table 2). The fresh roselle had also high levels of some fruity esters (methyl 3-methylbutanoate, methyl pentanoate, and 3-methylbutyl butanoate) and most of the detected aldehydes which among other are described as having green and citrus odors. This corresponds well with Ramirez-Rodrigues [26] who studied aroma profiles of hot and cold infusion of fresh and dried roselle.

All of the dried samples showed lower levels of the above-mentioned compounds, but significantly higher levels of five of the six furan compounds found. These compounds have odor descriptors like bread, almond, burnt sugar, and green/herbal and are often found in heat treated or roasted foods [27,28]. Chen et al. [29] found that thermal processing through air-drying produced caramel-like aroma, and this was also reported when drying roselle calyces [9,29,30]. Furans like furfural and 5-methyl-2-furfural can be formed through sugar degradation during heat treatment [28]. This formation can be accelerated by low moisture content (water activity 0.3–0.7 [31]). In our study, the dried samples had water activities in this range. Two terpenes (trans-linalooloxide and neroloxide) were highest in most of the dried samples, probably due to oxidation during drying [29].

Among the dried samples, the sun-dried differ by having significantly higher levels of certain ketones (2-heptanone, 2-octanone, 2-undecanone, 6-methyl-2-heptanone, 6-methyl-5-hepten-2-one, and 3-octen-2-one), esters (hexyl acetate, phenethyl acetate, methyl hexanoate, methyl octanoate and methyl nonanoate), and alcohols (pentanol and phenethyl alcohol). These compounds are not normally related to drying or heat treatment but rather indicate some degree of fermentation. This is most probably due to the earlier mentioned haze phenomenon, which made the sun drying slow and incomplete, and the findings for sun-dried samples in this study should therefore not be generalized.

### 2.3. Microstructure

**Figure 2:**
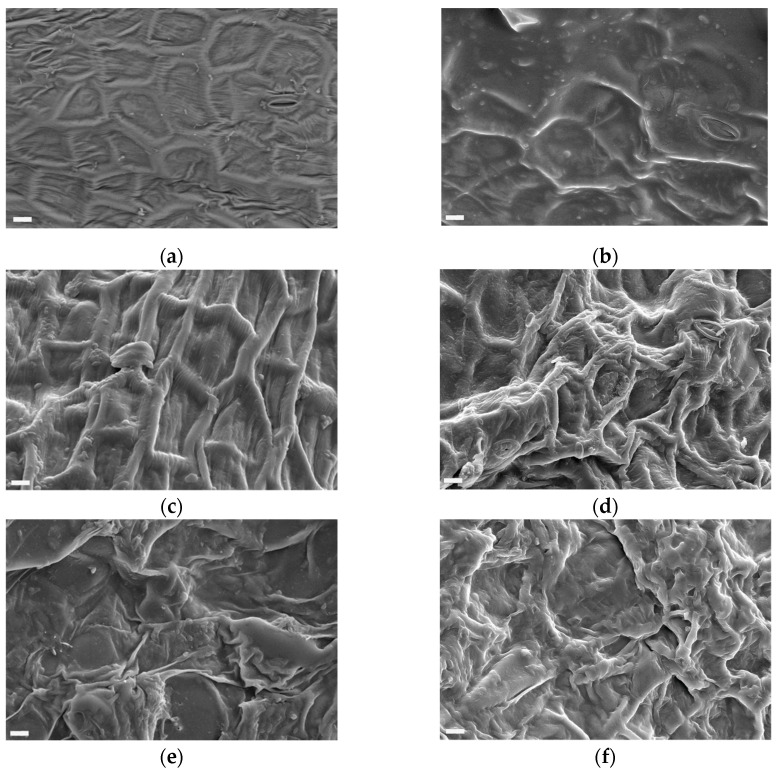
Scanning electron micrographs of the surface of roselle (*Hibiscus sabdariffa* L.) calyx samples after different treatments at a magnification of 500×. Bar = 10 µm: (**a**) fresh; (**b**) freeze-dried; (**c**) vacuum-dried; (**d**) commercial scale oven-dried; (**e**) lab scale oven-dried; (**f**) sun-dried.

Microstructural evaluation can elucidate product changes during the drying process and record how well-preserved the tissue is after the treatments. Figure 2 shows scanning electron micrographs of the surface of fresh and dried roselle calyx. The epidermis of the fresh calyx consists of thick-walled, tightly packed, and well-organized cells (Figure 2a), whereas the drying processes introduced various physical changes in the material (Figure 2b–f). The structural integrity of plant foods is mainly attributed to the primary cell wall, the middle lamella, and the turgor pressure generated within cells by osmosis. During most food processing operations, the turgor pressure is lost imparted by the disruption of the plasma membrane and vacuolar membrane, leading to cellular shrinkage.

However, no particular shrinkage was observed in the epidermis of the freeze-dried samples (Figure 2b). The surface was mostly smooth, the cell structure was well preserved and overall the sample appeared to have a more fresh-like quality. This is due to the fact that the samples were frozen before being dried, and the water was subsequently sublimated. The final structure of freeze-dried roselle is thus formed during freezing. This results in a porous structure, which will influence texture and dehydration capacity.

The microstructure of roselle was clearly affected by the vacuum-drying treatment (Figure 2c) as seen by the dehydrated, shrunken appearance of the epidermis cells. On the other hand, the cell layer appeared intact and individual cells could be identified as a result of this treatment. An explanation for the fairly well-preserved microstructure could be the formation of polymeric material resulting in increased thickness of the middle lamella between cells [32]. It was also observed that the texture of vacuum-dried roselle was more elastic and stretchable compared to the other dried samples.

Oven-drying extensively affected the roselle tissue structure and led to cell wall disruption, deformation, and folding (Figure 2d,e), probably introduced by the high drying temperature and velocity. This result is in agreement with other studies on apples [33,34]. The highest degree of tissue disruption and cell collapse was found in sun-dried roselle samples (Figure 2f) probably due to the long exposure.

The differences in microstructure may well explain some of the differences in color since a rough surface would be expected to have a lighter hue (lightness), while the original shape and structure (i.e., in fresh and freeze-dried) would intensify and deepen the color. This point is in accordance with Yousif et al. [35] who studied physical properties of vacuum-microwave-air-dried sweet basil. Further, the loss of turgor pressure during the drying processes will introduce color changes to the tissue, since the color of the roselle epidermis is due to water-soluble pigments located in the vacuole as well as lipid-soluble pigments located in the cytoplasm.

## 3. Materials and Methods

### 3.1. Sample Preparation

Fresh roselle (*Hibiscus sabdariffa* L.) calyx of the UMKL cultivar (obtained from HERBagus Sdn. Bhd, Kepala Batas, Malaysia) was chosen for the study. Samples used were harvested at a fully mature stage. The confirmation of plant species identification was based on taxonomic descriptions and photographic illustrations by botanist Dr Shamsul Khamis from the Institute of Bioscience (UPM Serdang, Malaysia). The fresh samples were manually sorted and washed thoroughly under running water to remove dirt and other extraneous matter. The excess water was drained and then the samples were weighed and kept in a chiller at 4 °C (less than 48 h) for further use.

### 3.2. Drying Experiments

Four different drying methods were compared in this study. Appropriate drying times and temperatures were determined in a preliminary experiment. The dried samples were kept in aluminum coated zip-lock packaging and stored at room temperature until further analysis. All drying experiments were performed in triplicate.

Oven-drying was carried out by (1) using a commercial scale oven (‘OVCOM’) in HERBagus Sdn. Bhd. Penang, Malaysia, and (2) using a laboratory scale oven (‘OVLAB’) in Faculty of Food Science and Technology, Universiti Putra Malaysia, Selangor, Malaysia. For commercial scale, a programmable oven model Box Oven 36 Tray (Kimah Industries Suppliers (M), Perai, Malaysia) was used.

**Figure 3:**
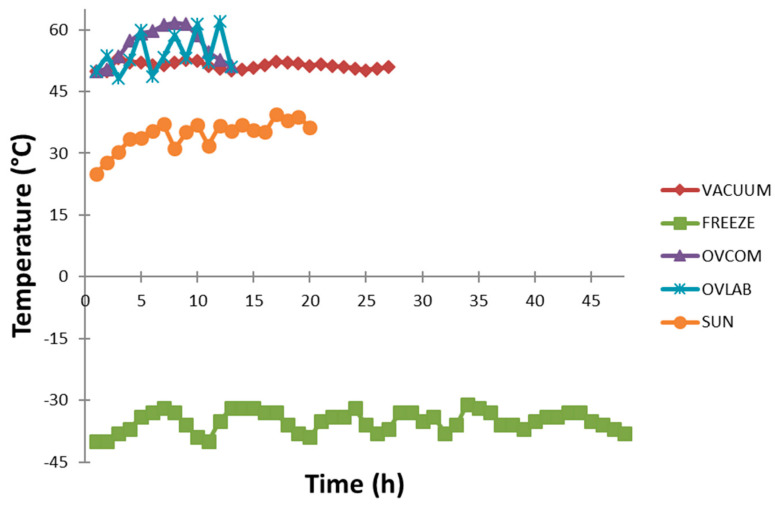
Drying temperature and time of the different methods applied.

The drying oven was equipped with an air ventilation outlet, temperature controller, and timer switch which allow the user to select the required drying temperature and adjust the time of drying. During the experiment, a perforated drying tray (90 cm × 58 cm) and (56 cm × 56 cm) (Kimah Industries Suppliers (M), Perai, Malaysia) was used. The air was circulated from the above and the bottom of the drying tray. For laboratory scale, fresh roselle calyx samples were dried on a perforated drying tray (73 cm × 65 cm) using a programmable oven 400W (Smoke Master Model SMA-112, Hanagi Seisakusho Co., Ltd., Toda, Japan). This oven was also equipped with a temperature control function and the air was circulated from the above and the bottom of the drying tray. The samples for both commercial and laboratory-scale were dried as a monolayer at 50 °C for 12 h at a constant air flow of 2 m s^−1^. The relative humidity of the ambient air (30 °C) was around 60–68%. During the drying process, the temperature of the drying air was recorded by a portable thermometer (EL-EnviroPad-TC, Corintech, Hampshire, United Kingdom) via an attached thermocouple probe (Figure 3). The oven was switched on for 30 min before the drying process to stabilize the temperature.

For freeze-drying (‘FREEZE’), samples were prepared as in 3.1 and then frozen in a freezer at −24 °C. Drying was carried out over 48 h in a freeze dryer (Freeze dry System LABCONCO Freezone 18^®^, Kansas City, MO, USA) at −40 °C and a pressure of 3.3 × 10^−3^ mbar.

Vacuum-drying (‘VACUUM’) was conducted using a vacuum-drying oven with a vacuum pump (Model VD 53, WTB Binder, Berlin, Germany) at 50 °C for 26 h at a pressure of 28 mbar. Then, 400 g of sample were spread as a monolayer on the aluminum expansion racks and were dried in the vacuum chamber. The temperature was recorded as described at oven-drying. The oven was switched on for 30 min before the drying process to stabilize the temperature.

Sun-drying (‘SUN’) was carried out in a monolayer on a tray directly under the hot sun (<45 °C) for 19 h. The temperature was recorded as above. The relative humidity during sun drying was around 59–70%.

### 3.3. Physicochemical Analysis

Fresh and dried roselle calyx samples were ground for 2 min with high speed using a blender (Panasonic MX-M1011, Petaling Jaya, Malaysia) and mixed thoroughly. The ground samples were kept in aluminum coated zip-lock packages and stored at room temperature until further analysis. Moisture content was determined in triplicate using an air-oven method at 105 °C until constant weight [36]. The water activity was measured using a water activity meter (Aqualab Series 3 TE, Decagon Device, Inc, Pullman, WA, USA). Samples were measured in triplicate at 25 ± 1 °C. The samples were placed in a glass petri dish (7.4 cm diameter) and read by a Minolta Chroma Meter CR-300 Series 2° (Konica Minolta Sensing Americas, Inc., NJ, USA) observer through the bottom of the petri dish. The colorimeter was calibrated using a standard Minolta calibration plate. Triplicate measurements were recorded using the L*, a*, b* system.

An extract was prepared from 4 g of ground roselle mixed with 40 mL of tap water and soaked for 30 min. The extract was filtered using filter paper (S&S folded filters, Schleicher & Schuell, Chicago, IL, USA, 320 mm). Total soluble solids were determined in triplicate using an LCD Digital Bench Model Refractometer (HI96801, Hanna Instrument Inc., Nusfalau, Romania) and color was measured as described above.

### 3.4. Volatile Compounds

All analyses were carried out in triplicate as reported by Juhari et al. [30]. Whole roselle (fresh or dry) was ground for 2 min using a blender (KRUPS Speedy PRO, Group SEB Nordic AS, Ballerup, Denmark), and 10 g of ground roselle was mixed with 40 mL of tap water. The compound 4-methyl-1-pentanol (1 mL of a 5 mg L^−1^ solution) was added as internal standard. Each sample was placed in a gas washing flask (300 mL, 7.5 cm diameter) together with a magnetic stirrer to agitate the sample during volatile extraction. Volatile compounds were collected on a Tenax-TA trap connected to the flask’s outlet. The trap contained 200 mg of Tenax-TA mesh size 60/80, density of 0.37 g mL^−1^, Buchem bv, Apeldoorn, The Netherlands. The samples were equilibrated to 40 °C ± 1 °C in a circulating water bath and then purged with nitrogen (100 mL min^−1^) for 30 min. Water was removed from the traps using a flow of dry nitrogen (100 mL min^−1^ for 10 min). The Tenax-TA traps were then capped and stored at 5 °C before analysis by gas chromatography-mass spectrometry.

The trapped volatiles were desorbed using an automatic thermal desorption unit (TurboMatrix 350, Perkin Elmer, Shelton, CT, USA). Primary desorption was carried out by heating the trap to 250 °C with a flow (50 mL min^−1^) of carrier gas (H_2_) for 15.0 min. The stripped volatiles were trapped in a Tenax TA cold trap (30 mg held at 5 °C), which was subsequently heated at 300 °C for 4 min (secondary desorption, outlet split 1:10). This allowed for rapid transfer of volatiles to a gas chromatograph-mass spectrometer (GC-MS, 7890A GC-system interfaced with a 5975C VL MSD with Triple-Axis detector from Agilent Technologies, Palo Alto, CA, USA) through a heated (225 °C) transfer line. Separation of volatiles was carried out on a DB-Wax capillary column (30 m long × 0.25 mm internal diameter, 0.50 µm film thickness). The column pressure was held constant at 2.4 psi resulting in an initial flow rate of 1.4 mL min^−1^ using hydrogen as carrier gas. The column temperature program was: 10 min at 30 °C, from 30 °C to 240 °C at 8 °C min^−1^, and finally 5 min at 240 °C. The mass spectrometer was operating in the electron ionization mode at 70 eV. Mass-to-charge ratios between 15 and 300 were scanned. Volatile compounds were identified by probability-based matching of their mass spectra with those of a commercial database (Wiley275.L, HP Product no. G1035A) using the software program, MSDChemstation (Version E.02.00, Agilent Technologies, Palo Alto, CA, USA). Volatile compound identification was confirmed by comparison with retention indices (RI) of authentic reference compounds or retention indices in the literature. Since the study included fresh as well as dried roselle calyces, the results from volatile analyses are presented as peak areas relative to dry matter content.

### 3.5. Microstructural Analysis

Tissue pieces were arranged on aluminum stubs on double-sided tape with the epidermis upwards, and coated with a layer of gold using a Sputter Coater SCD 005 (Bal-Tec Company, Buffalo Grove, IL, USA) for the dried roselle calyx, whereas no coating of a sample was done on fresh roselle calyx. The dried samples were examined using secondary electron (SE) detector while the fresh samples were examined using backscattered electron (QBSD) detector in a scanning electron microscope, SEM (Leo 1455VP Variable Pressure, Cambridge, UK) operating at an accelerating voltage of 20 kV. Micrographs were taken at a magnification of 100×, 500× and 1000×. The microstructural analysis was carried out in triplicate.

### 3.6. Data Analysis

All data from the physicochemical analyses and volatile compound analysis were analyzed by one-way analysis of variance (ANOVA) using the software JMP 13 (SAS Institute Inc, Cary, NC, USA). Post hoc calculations using Student’s *t*-test were used for multiple comparisons. Multivariate analysis was applied to GC-MS data to evaluate the variation between the fresh and dried roselle samples. PCA is a multivariate projection method designed to extract and visually display the systematic variation in the data matrix of the volatile compounds, making it possible to include many statistical variables at the same time. PCA was performed using the Latentix software (LatentiX 2.0, Latent5, Copenhagen, Denmark). Analyses were carried out on the average of significant (*p* < 0.05) peak areas and the data were auto-scaled.

## 4. Conclusions

All the tested drying methods, except sun-drying, reduced moisture content and water activity to a safe level (water activity = 0.605 or lower). When extracts were prepared, it turned out that the extract based on freeze-dried roselle had the highest value of TSS. Furthermore, the color after vacuum-drying was the one closest to the color of fresh samples, both when evaluated in ground and liquid form. All drying methods reduced terpenes, some aldehydes, and esters which are expected to contribute to typical fruity, floral, spicy, and green odors. At the same time furan compounds increased. These compounds are most likely causing the caramel-like aroma that has been reported in dried roselle. Among the dried samples, the sun-dried stood out as having higher levels of some ketones, alcohols, and esters. This was possibly due to unintended fermentation processes. Drying was found to significantly affect microstructure, but freeze-drying preserved the cell structure best. The different drying methods applied had different effects on quality, and the present study may therefore serve as a tool for the choice of the most appropriate drying techniques to be used for the production and further commercialization of dried roselle calyx.

## Figures and Tables

**Table 1:** Physicochemical properties in the fresh and dried roselle (*Hibiscus sabdariffa* L.) calyx and filtrate.

			FRESH	VACUUM	FREEZE	OVCOM	OVLAB	SUN	Significance
Roselle calyx	Moisture content (%)		89.4 ± 0.1 ^a^	12.9 ± 0.1 ^c^	9.6 ± 0.2 ^d^	9.5 ± 1.4 ^d^	8.9 ± 0 ^d^	15.6 ± 0.1 ^b^	***
	Water activity (A_w_)		1.000 ± 0 ^a^	0.605 ± 0 ^c^	0.414 ± 0 ^e^	0.482 ± 0 ^d^	0.395 ± 0 ^e^	0.727 ± 0 ^b^	***
	Color value -measured in ground form	L*	34.7 ± 0.4 ^b^	35.2 ± 1.1 ^b^	38.9 ± 0.3 ^a^	39.2 ± 0.4 ^a^	39.1 ± 0.4 ^a^	38.8 ± 0.5 ^a^	***
		a*	4.5 ± 0.3 ^d^	4.5 ± 1.0 ^d^	11.8 ± 0.4 ^a^	10.5 ± 0.2 ^b^	7.2 ± 0.6 ^c^	4.8 ± 0.4 ^d^	***
		b*	5.9 ± 0 ^bc^	6.4 ± 0.5 ^b^	7.7 ± 0.1 ^a^	7.8 ± 0.2 ^a^	5.8 ± 0.1 ^c^	8.2 ± 0.5 ^a^	***
Filtrate	Total soluble solids (%)		0.4 ± 0.1 ^f^	1.1 ± 0.1 ^e^	3.8 ± 0 ^a^	3.5 ± 0 ^b^	3.1 ± 0.2 ^c^	2.8 ± 0 ^d^	***
	Color value -measured in liquid form	L*	43.1 ± 0 ^b^	45.0 ± 0.1 ^a^	35.6 ± 0.1 ^d^	35.5 ± 0.1 ^d^	35.6 ± 0.2 ^d^	36.2 ± 0 ^c^	***
		a*	20.9 ± 0.2 ^a^	16.2 ± 0.2 ^b^	6.3 ± 0.1 ^e^	7.1 ± 0 ^d^	6.1 ± 0.1 ^e^	8.8 ± 0.1 ^c^	***
		b*	17.5 ± 0.2 ^a^	15.6 ± 0.1 ^b^	5.9 ± 0 ^d^	6.1 ± 0.1 ^d^	6.0 ± 0 ^d^	7.1 ± 0 ^c^	***

Values in a row not marked with the same letter are significantly different, Student’s *t*-test (*p* < 0.05). *** Indicates significance at *p* < 0.001. FRESH = Fresh roselle calyx; VACUUM = Vacuum-dried roselle calyx; FREEZE = Freeze-dried roselle calyx; OVCOM = commercial scale oven-dried roselle calyx; OVLAB = lab scale oven-dried roselle calyx; SUN = sun-dried roselle calyx.

**Table 2:** Volatile compounds (peak areas relative to dry matter content) identified in the fresh and dried roselle (*Hibiscus sabdariffa* L.) calyx using different drying methods.

Compounds	Calculated RI ^a^	Reference RI	ID ^b^	Odor Description	FRESH	VACUUM	FREEZE	OVCOM	OVLAB	SUN	Significance ^g^
**Terpenes**											
*β*-Myrcene	1154	1170	Standard	Musty, fruity, lemon, spice, woody ^d^	10534	989	542	0	683	0	***
Limonene	1179	1200	Standard	Citrus, fruity, green ^d^	39828	4057	9728	9807	11478	5197	***
1,8-Cineole	1187	1193	Standard	Camphor, minty, pine, liquorice, mentholic ^d^	16625	869	421	1003	458	0	***
*p*-Cymene	1256	1261	Standard	Lemon, fruity, sweet, herbal ^d^	15767	2188	36723	34849	30681	4158	*
*α*-Terpinolene	1270	1297	Standard	Woody, fruity, sweet, pine ^d^	11089	1227	4305	4218	4825	1415	***
Isoterpinolene	1385	1331	Literature	Woody, pine, citrus ^e^	4447	1104	920	6762	3073	4642	***
*trans*-Linalool oxide	1426	1438	Literature	Floral, creamy, earthy, green ^c^	321	5711	3316	8425	0	5514	*
Neroloxide	1460	1485	Standard	Oily, flowery ^d^	1280	0	814	3038	1777	0	*
Camphor	1509	1498	Literature	Camphor, green, leafy	3211	1477	833	7072	3582	3502	n.s.
Linalool	1528	1534	Standard	Citrus, herbal, flowery ^c^	548431	6071	5819	9672	17309	22827	***
*α*-isophorone	1583	1591	Literature	Woody, champor, musty ^e^	9145	376	2461	1185	0	1403	***
1-p-menthen-9-al	1602	1620	Literature	Spice, herbal ^d^	647	3897	3007	7325	4224	7277	n.s.
Aromadendrene	1627	1628	Standard	Sweet, dry ^d^	2063	0	976	0	2126	0	n.s.
*α*-Bisabolene	1653	1702	Literature	Berry, spicy, citrus ^d^	7398	0	433	0	370	0	***
*α*-Terpineol	1683	1682	Literature	Pine, lillac, woody, floral ^e^	31865	2742	5374	7928	6826	10288	***
Azulene	1724	1746	Literature	Medicinal ^d^	35528	4193	2433	3614	28092	7563	***
*δ*-Cadinene	1735	1749	Literature	Thyme, medicinal, woody ^c^	15947	1516	1944	1798	2683	2979	***
*α*-Calacorene	1896	1904	Literature	Woody ^c^	1104	90	452	261	520	358	***
**Esters** **Ethyl Ester**											
Ethyl acetate	864	867	Standard	Pineapple ^c^, fruity ^d^	164108	168821	5750	63801	188847	48039	n.s.
**Acetate Esters**											
Methyl acetate	807	810	Literature	Fruity, solvent-like ^d^	14252	442728	13023	243853	132356	290587	**
2-Methylpropyl acetate	999	1017	Standard	Fruity, flowery, strong, banana, pear ^d^	1631	17130	0	1005	1905	0	***
3-Methylbutyl acetate	1111	1112	Literature	Sweet, banana, fruity, green ^e^	602	29269	715	1998	5342	21192	**
Pentyl acetate	1165	1172	Literature	Herbal ^d^	1834	1415	0	1772	658	8173	n.s.
Hexyl acetate	1266	1293	Standard	Fruity, Herbal ^c^	1041	1140	0	0	0	13768	*
Phenethyl acetate	1797	1795	Standard	Rose, floral, fruity, sweet ^d^	0	2889	0	0	0	17139	**
**Other esters**											
Methyl 2-methylpropanoate	907	910	Literature	Fruity, floral ^e^	3557	10362	906	736	783	0	***
Methyl 2-methylbutanoate	991	1000	Literature	Apple, fruity ^c^	799	6133	0	0	0	0	***
Methyl 3-methylbutanoate	1003	1011	Literature	Fruity, apple ^d^	13640	4149	12	0	120	0	***
Methyl pentanoate	1076	1086	Literature	Sweet, ethereal, apple ^d^	1606	0	0	397	101	0	*
Methyl hexanoate	1176	1196	Standard	Fruity, fresh, sweet ^c^	1515	1691	213	3057	662	17626	*
3-Methylbutyl butanoate	1236	1256	Literature	Fruity, apple, spicy, buttery ^e^	40922	0	0	1709	0	0	***
Methyl octanoate	1375	1401	Standard	Orange ^c^	0	299	174	586	466	4871	**
Methyl nonanoate	1471	1481	Literature	Coconut ^c^	0	0	87	847	377	1622	***
Methyl salicylate	1759	1797	Standard	Peppermint ^c^	602	447	176	1020	356	480	n.s.
**Aldehydes**											
2-Methylpropanal	787	789	Literature	Green, pungent, burnt, malty ^d^	0	11900	5142	7050	12196	6909	*
2-Methylbutanal	893	896	Standard	Cocoa, almond, malty^c^ fermented ^f^	33135	22471	4677	13602	20546	8145	*
3-Methylbutanal	900	917	Standard	Fruity, almond, toasted, malty, green ^d^	14846	37241	5803	12035	29298	22709	n.s.
Pentanal	948	968	Standard	Almond, malty, pungent ^d^	46310	8401	7752	22431	29236	20175	n.s.
Hexanal	1068	1087	Standard	Grassy ^c^	100121	5753	16053	59056	27115	46659	***
Heptanal	1172	1192	Standard	Fatty, citrus, rancid ^c^	15731	946	1235	6669	3076	5293	***
2-Hexenal	1196	1205	Literature	Apple, green, leaf ^c^	14741	0	3260	1753	2056	1767	***
Octanal	1279	1311	Standard	Orange peel, pungent ^f^	20491	772	1263	4275	2202	6123	**
Nonanal	1379	1402	Standard	Fatty, citrus, green ^c^	75446	3183	5419	23189	12695	20012	***
Decanal	1480	1511	Standard	Green, waxy, floral, tallow ^d^	5368	379	388	1733	0	2799	*
Benzaldehyde	1503	1537	Standard	Almond ^c^	53396	22030	8172	11396	30133	49355	*
Phenylethanal	1622	1636	Literature	Honey, sweet ^c^	2733	3822	1344	2384	2867	4030	n.s.
**Ketones**											
2-Butanone	877	881	Standard	Ether-like ^d^, fruity ^e^	100594	13176	2816	5570	9444	8845	***
2-Pentanone	1103	1023	Literature	Fruity, wine, woody ^e^	318	9732	0	3618	5058	0	n.s.
4-Methyl-3-penten-2-one	1112	1113	Literature	Minty ^d^	378	1054	388	2804	1936	1599	**
2-Heptanone	1168	1189	Standard	Soap^c^, blue cheese ^f^	6000	613	69	2061	712	6591	***
6-Methyl-2-heptanone	1223	1228	Literature	Camphoreous ^e^	3796	547	190	1666	1397	5859	**
2-Octanone	1276	1283	Literature	Earthy, woody, herbal, yeasty ^e^	2081	230	0	1204	0	15423	*
2,6,6-Trimetylcyclohexanone	1304	1333	Literature	Pungent ^d^, honey, citrus ^f^	3322	641	414	3570	1345	3164	***
6-Methyl-5-hepten-2-one	1329	1339	Standard	Mushroom, earthy, woody, rubbery ^d^	17970	3421	4008	12375	7464	26764	***
3-Octen-2-one	1381	1392	Literature	Earthy, spicy, herbal ^e^	327	0	0	0	0	2768	***
2-Undecanone	1579	1580	Literature	Waxy, fruity, pineapple ^e^	551	0	0	440	256	2597	***
1-Phenylethanone	1632	1645	Literature	Almond, floral ^d^, musty ^c^	6241	2515	1165	2683	2196	3052	***
**Furans**											
2-Methylfuran	865	877	Literature	Ether-like, chocolate ^d^	478	929	0	932	821	1317	n.s.
2-Pentylfuran	1218	1229	Literature	Green bean ^c^, pungent ^f^	6330	2120	1877	31274	6815	53767	***
5-Isopropenyl-2-methyl-2-vinyltetrahydrofuran	1227	1253	MS	Fresh, forest, grassy ^c^	407	3770	3579	25511	9142	4485	***
Furfural	1443	1458	Standard	Bread, almond ^c^	5093	379572	185077	565607	442585	380159	***
2-Acetylfuran	1486	1497	Standard	Balsamic ^c^	606	22074	6933	35757	29182	32601	***
5-Methyl-2-furfural	1554	1560	Standard	Almond, caramel, burnt sugar ^c^	1710	9757	2555	53826	23679	24234	***
**Alcohols**											
2-Methyl-1-propanol	1089	1100	Standard	Etherial, whiney ^e^	765	12669	497	619	4286	9570	***
3-methylbutanol	1199	1222	Standard	Fusel, pungent, etherial, banana ^e^	50370	29394	576	6440	20607	33726	***
1-Pentanol	1248	1274	Standard	Alcohol, pungent, fruity, balsamic ^c^	6211	1651	935	2987	2475	10508	**
1-Hexanol	1348	1372	Standard	Resin, flowery, green ^c^	118601	3188	1663	0	3238	72258	***
1-Nonanol	1639	1640	Literature	Fatty, green ^c^	915	0	0	0	0	3752	***
Phenethyl alcohol	1894	1932	Standard	Honey, spice, rose, lilac ^c^	0	3761	0	0	585	5563	***
**Phenols**											
*p*-Cresol	1884	1902	Literature	Medicinal, phenol, smoke ^c^	2201	1163	4553	6744	6671	8057	n.s.
Phenol	1976	1987	Literature	Phenolic, medicinal ^d^	6872	1163	609	826	499	762	***
*p*-Ethylguaiacol	2005	2008	Literature	Spice, clove ^c^	0	329	1707	646	1459	2854	*
**Acid**											
Pentanoic acid	1647	1685	Literature	Sweaty, pungent, sour, cheesy, beefy ^d^	0	744	0	0	0	0	*
**Lactone**											
*γ*-Butyrolactone	1610	1617	Literature	Caramel, sweet ^c^	461	2709	305	1072	1037	2902	**

^a^ The retention indices (RIs) of volatiles were calculated as the retention time of the volatiles normalized to the retention times of adjacently eluting n-alkanes (C6-C22); ^b^ Identification method: ‘Standard’: retention time and mass spectrum confirmed by authentic standard run on the same system, ‘Literature’: linear retention index is close to retention indices from Flavornet/Pherobase/NIST Webbook/PubChem for DB-wax capillary GC column, ‘MS’: mass spectrum agrees with mass spectrum in database; ^c^ Odor description based on Flavornet; ^d^ Odor description based on Pherobase; ^e^ Odor description based on The Good Scents Company; ^f^ Odor description based on Odor.org.uk; *,**,*** indicate significance at *p* < 0.05, *p* < 0.01, and *p* < 0.001, respectively; n.s. means no significant difference between the samples.

## Data Availability

Not applicable.

## Sample Availability

Samples of the compounds are not available from the authors.

## References

[1] Mamat M.R. (2016). 2 Villages of Roselle.

[2] Husin N.N. (2010). Roselle Plant Has High Demand for Export to Australia.

[3] Villani T., Juliani H.R., Simon J.E., Wu Q.L. (2013). *Hibiscus sabdariffa*: Phytochemistry, Quality Control, and Health Properties. ACS Symp. Ser..

[4] Ibrahim R., Mazuki N.A.F. (2013). The quality of roselle (*Hibiscus sabdariffa* L.) juices made from roselle calyces stored at different cold temperatures. Malays. Appl. Biol..

[5] Sabet Sarvestani S., Hosseini S.M., Farhangfar S.H. (2020). Effect of aqueous-alcoholic extract of *Hibiscus sabdariffa* calyx and leaf calyx and leaf on performance, egg quality, immune system and antioxidant balance of laying hens. Iran. J. Appl. Anim. Sci..

[6] Shruthi V.H., Ramachandra C.T. (2019). Roselle (Hibiscus sabdariffa L.) Calyces: A Potential Source of Natural Color and Its Health Benefits. Food Bioactives.

[7] Garg H., Kumar R. Development in Solar Drying. Proceedings of the 2nd Asian-Oceania Drying Conference (ADC 2001).

[8] Sulieman A.M. (2014). Spray drying of karkade (*Hibiscus sabdariffa* L.) calyces and evaluation of the product. Int. J. Food Eng..

[9] Builders P.F., Ezeobi C.R., Tarfa F.D., Builders M.I. (2010). Assessment of the intrinsic and stability properties of the freeze-dried and formulated extract of *Hibiscus sabdariffa* Linn. (Malvaceae). African J. Pharm. Pharmacol..

[10] Meza-Jimenez J., Ramirez-Ruiz J., Diaz-Nunes J. (2008). The design and proposal of a thermodynamic drying system for the dehydration of Roselle (*Hibiscus Sabdariffa*) and other agro-industrial products. African J. Agric. Res..

[11] Daniel D.L., Huerta B.B., Sosa I.A., Mendoza M.V. (2012). Effect of fixed bed drying on the retention of phenolic compounds, anthocyanins and antioxidant activity of roselle (*Hibiscus sabdariffa* L.). Ind. Crops Prod..

[12] Zaman H.U., Das P., Das P., Sahu N.K. (2017). Analysis of physicochemical, nutritional and antioxidant properties of fresh and dried Roselle (*Hibiscus sabdariffa* Linn.) calyces. Int. J. Pure App. Biosci.

[13] Gonzalez-Palomares S., Estarrón-Espinosa M., Gómez-Leyva J.F., Andrade-González I. (2009). Effect of the temperature on the spray drying of Roselle extracts (*Hibiscus sabdariffa* L.). Plant Foods Hum. Nutr..

[14] Aghbashlo M., Kianmehr M.H., Samimi-Akhijahani H. (2008). Influence of drying conditions on the effective moisture diffusivity, energy of activation and energy consumption during the thin-layer drying of berberis fruit (Berberidaceae). Energy Convers. Manag..

[15] Lewicki P.P. (2006). Design of hot air drying for better foods. Trends Food Sci. Technol..

[16] Agudelo C., Barros L., Santos-Buelga C., Martínez-Navarrete N., Ferreira I.C.F.R. (2017). Phytochemical content and antioxidant activity of grapefruit (Star Ruby): A comparison between fresh freeze-dried fruits and different powder formulations. LWT-Food Sci. Technol..

[17] Martínez-Navarrete N., Salvador A., Oliva C., Camacho M.M. (2019). Influence of biopolymenrs and freeze-drying shelf temperature on the quality of a mandarin snack. LWT.

[18] Fontana A. Water Activity: Why it is Important for Food Safety?. Proceedings of the NSP International Conference on Food Safety.

[19] Decagon Device I. Water Activity for Product Safety and Quality. http://www.aqualab.com/education/water-activity-for-product-safety-and-quality/.

[20] Okanlawon S., Ibrahim M., Oyebanji A. (2002). Effect of pre-drying treatment on the storage of dried tomatoes. Trop. Sci..

[21] Ratti C. (2001). Hot air and freeze-drying of high-value foods: A review. J. Food Eng..

[22] Juhari N.H., Lasekan O., Kharidah M., Ab Karim S. (2012). Optimization of hot-air drying conditions on the physicochemical characteristics of torch ginger (*Etlingera elatior*). J. Food, Agric. Environ..

[23] Prachayawarakorn S., Prachayawasin P., Soponronnarit S. (2004). Effective diffusivity and kinetics of urease inactivation and color change during processing of soybeans with superheated-steam fluidized bed. Dry. Technol..

[24] Wu H.Y., Yang K.M., Chiang P.Y. (2018). Roselle anthocyanins: Antioxidant properties and stability to heat and pH. Molecules.

[25] Calín-Sánchez Á., Lipan L., Cano-Lamadrid M., Kharaghani A., Masztalerz K., Carbonell-Barrachina A.A., Figiel A. (2020). Comparison of traditional and novel drying techniques and its effect on quality of fruits, vegetables and aromatic herbs. Foods.

[26] Ramírez-Rodrigues M., Balaban M., Marshall M., Rouseff R. (2011). Hot and cold water infusion aroma profiles of *Hibiscus sabdariffa*: Fresh compared with dried. J. Food Sci..

[27] Belitz H.D., Grosch W., Schieberle P. (2009). Aroma compounds. Food Chemistry.

[28] Kroh L.W. (1994). Caramelisation in food and beverages. Food Chem..

[29] Chen S., Huang T.C., Ho C.T., Tsai P.J. (1998). Extraction, analysis, and study on the volatiles in roselle tea. J. Agric. Food Chem..

[30] Juhari N.H., Varming C., Petersen M.A., Andrews J., Taylor D.S.M. (2014). Analysis of Aroma Compounds of Roselle by Dynamic Headspace Sampling using Different Sample Preparation Methods. XIV Weurman Flavour Research Symposium.

[31] Eichnerl K., Karel M. (1972). The influence of water content and water activity on the sugar-amino browning reaction in model systems under various conditions. J. Agric. Food Chem..

[32] Deng Y., Zhao Y. (2008). Effects of pulsed-vacuum and ultrasound on the osmodehydration kinetics and microstructure of apples (Fuji). J. Food Eng..

[33] Deng Y., Zhao Y. (2008). Effect of pulsed vacuum and ultrasound osmopretreatments on glass transition temperature, texture, microstructure and calcium penetration of dried apples (Fuji). LWT-Food Sci. Technol..

[34] Lewicki P.P., Pawlak G. (2003). Effect of drying on microstructure of plant tissue. Dry. Technol..

[35] Yousif A.N., Scaman C.H., Durance T.D., Girard B. (1999). Flavor volatiles and physical properties of vacuum-microwave- and air-dried sweet basil (*Ocimum basilicum* L.). J. Agric. Food Chem..

[36] (1984). AOAC Official Methods of Analysis of the Association of Official Analytical Chemists.

